# Mechanism of GSDMD in the Pathogenesis of *Pasteurella multocida* PmCQ2

**DOI:** 10.1155/tbed/4436022

**Published:** 2026-02-11

**Authors:** Qingqing Yang, Wei Wang, Xin Shen, Yi Lu, Jiajia Zheng, Jinrong Ran, Xuefeng Cao, Rendong Fang

**Affiliations:** ^1^ Joint International Research Laboratory of Animal Health and Animal Food Safety, College of Veterinary Medicine, Southwest University, Chongqing, 400715, China, swu.edu.cn

**Keywords:** GSDMD, NLRP3, *Pasteurella multocida*, pyroptosis

## Abstract

*Pasteurella multocida* serotype A (PmCQ2), a Gram‐negative zoonotic pathogen, causes severe respiratory disease in a variety of domestic and wild animals, leading to high morbidity and mortality and substantial agricultural economic losses. Pyroptosis, a gasdermin‐mediated programmed cell death mechanism, facilitates pathogen clearance but exacerbates tissue damage through inflammatory cytokine release. While our prior work established PmCQ2‐driven NOD‐like receptor thermal protein domain associated protein 3 (NLRP3) inflammasome activation, the role of pyroptosis in pulmonary pathology during infection remains unresolved. Here, we demonstrate that PmCQ2 induces macrophage pyroptosis via gasdermin D (GSDMD) cleavage, evidenced by lactate dehydrogenase (LDH) release, membrane pore formation under transmission electron microscopy (TEM), and proteolytic generation of GSDMD‐N termini. Pharmacological inhibition of NLRP3 (MCC950) and genetic ablation of caspase‐11 significantly attenuated GSDMD activation, IL‐1β secretion, and pyroptotic cell death, implicating both canonical (NLRP3/caspase‐1) and noncanonical (caspase‐11) pathways. Crucially, GSDMD knockout mice exhibited markedly reduced lung injury, evidenced by diminished inflammatory infiltration and preserved alveolar architecture, compared to wild‐type (WT) counterparts following PmCQ2 challenge. This study provides the first evidence that PmCQ2 triggers GSDMD‐dependent pyroptosis through dual signaling axes, directly linking this inflammatory cell death pathway to pathogen‐induced pulmonary damage. Our findings position GSDMD as a central therapeutic target to mitigate tissue injury during *P. multocida* infection, offering a framework for novel interventions that balance antimicrobial defense and inflammation control in zoonotic pathogens.

## 1. Introduction

*Pasteurella multocida*, a Gram‐negative pathogen of veterinary and zoonotic significance, causes severe diseases such as hemorrhagic septicemia, atrophic rhinitis, and respiratory infections across diverse hosts [1]. Among its serotypes, type A strains like CQ2 (PmCQ2) exhibit heightened virulence, driving invasive pulmonary infections and systemic inflammatory injury with high mortality [2]. Current therapeutic strategies remain limited by antibiotic resistance and incomplete mechanistic insights into host–pathogen interactions, underscoring the need to dissect molecular drivers of *P. multocida*‐induced immunopathology [3].

Innate immunity combats pathogens through pattern recognition receptors (PRRs) such as NOD‐like receptor thermal protein domain associated protein 3 (NLRP3), which detects microbial threats and orchestrates inflammasome assembly [4, 5]. Activated NLRP3 recruits caspase recruitment domain (ASC) and caspase‐1, triggering proteolytic maturation of IL‐1β/IL‐18 and GSDMD‐mediated pyroptosis—a lytic cell death that amplifies inflammation [6]. GSDMD, a pore‐forming executor of pyroptosis, is cleaved by caspase‐1/11 to release its N‐terminal domain (GSDMD‐NT), which oligomerizes to permeabilize membranes, releasing cytokines and propagating immune responses [7]. Recent studies reveal nuanced regulatory mechanisms, including S‐palmitoylation modifications that spatially control GSDMD activation, and noncanonical pathways where full‐length GSDME induces pyroptosis independent of proteolytic cleavage [8, 9]. These discoveries highlight the complexity of gasdermins biology and its therapeutic potential in inflammatory diseases [10].

Accumulating evidence indicates that diverse pathogens hijack GSDMD signaling to trigger pyroptosis, thereby exacerbating tissue injury. For instance, *Staphylococcus aureus* activates the NLRP3 inflammasome via pore‐forming toxins, driving caspase‐1‐dependent cell death, whereas *Escherichia coli* triggers the noncanonical caspase‐11 axis through cytosolic lipopolysaccharide (LPS) sensing [11, 12]. Similarly, the Gram‐negative pathogen *Klebsiella pneumoniae* activates caspase‐11‐mediated pyroptosis, thereby contributing to sepsis‐related organ damage [13]. More recently, SARS‐CoV‐2 has been shown to aggravate pulmonary pathology and cytokine storm syndromes specifically through GSDMD cleavage [14]. Mechanistically, this pathogenicity is driven by excessive GSDMD‐mediated pore formation, which triggers widespread lytic cell death and an uncontrolled release of pro‐inflammatory cytokines. In contrast, *Shigella flexneri* has evolved an immune evasion strategy by secreting the E3 ubiquitin ligase IpaH7.8, which targets GSDMD for degradation and thereby suppresses pyroptosis [15]. Pharmacological inhibition of NLRP3 (MCC950), caspases (Z‐VAD‐FMK), potassium efflux (KCl), or reactive oxygen species (ROS; N‐Acetylcysteine [NAC]) [15] attenuates pyroptosis, implicating canonical pathways [16–18]. However, the role of GSDMD in *P. multocida* pathogenesis remains enigmatic. Unlike pathogens where GSDMD serves primarily as a clearance effector or driver of septic shock, it is unclear whether *P. multocida* uniquely exploits GSDMD‐mediated membrane dynamics to facilitate invasion, marking a distinct pathogenic mechanism.

Emerging evidence positions GSDMD as a dual regulator of cell death and inflammation. In chronic oxalate nephropathy, GSDMD deficiency exacerbates renal injury by shifting cell death to necroptosis, revealing cross talk between pyroptotic and necroptotic pathways [16]. Similarly, GSDMD promotes Parkinson’s disease progression by activating microglia and dopaminergic neuron death, highlighting its context‐dependent roles [9]. Recent advancements in GSDMD modulation offer translational promise. Small‐molecule agonists like 6,7‐dichloro‐2‐methylsulfonyl‐3‐N‐tert‐butylaminoquinoxaline (DMB) activate GSDMD pores without proteolysis, enhancing antitumor immunity, while palmitoylation inhibitors mitigate septic shock by blocking GSDMD membrane translocation [8, 10]. Such strategies could be adapted to temper hyperinflammation while preserving host defense.

In this study, we delineate GSDMD’s centrality in PmCQ2 pathogenesis. By integrating molecular, cellular, and animal models, we establish that PmCQ2 exploits NLRP3‐GSDMD signaling to drive pyroptosis and systemic inflammation. Our work bridges mechanistic gaps in *P. multocida* immunobiology and positions GSDMD as a therapeutic node for curbing bacterial virulence and immunopathology.

## 2. Materials and Methods

### 2.1. Mice

Wild‐type (WT) C57BL/6 mice were obtained from Chongqing Academy of Chinese Material Medical (Chongqing, China). *Gsdmd* ^*−/−*^ mice were kindly gifted by Dr. Feng Shao from the NIBS (National Institute of Biological Sciences, Beijing, China). These knockout mice were on a C57BL/6 background and maintained in specific pathogen free (SPF) conditions for being used at 8–10 weeks of age. All animal procedures were conducted in compliance with institutional guidelines and approved by the Institutional Animal Care and Use Committee (IACUC) of Southwest University, Chongqing, China (Protocol Number IACUC‐20221022‐03).

### 2.2. Bacterial Strains

Bovine origin capsule type A *P. multocida* PmCQ2 (GenBank Entry Number: LIUN00000000) was isolated from the lungs of a calf with pneumonia in Chongqing and stored at −80°C. The bacteria were cultured on a Martin AGAR plate at 37°C for 18–24 h. A single colony was selected and inoculated in 5 mL Martin Broth (Solarbio, China) supplemented with 5% fetal calf serum (FCS, Gibco, USA) at 37°C for 12 h. Bacterial concentrations were quantified via viable colony counts, and cultures were diluted in cell culture medium to the required multiplicity of infection (MOI) for subsequent assays.

### 2.3. Preparation of Peritoneal Macrophages (PECs) and *P. multocida* Infection *In Vitro*

Primary mouse PECs were aspirated as described previously [17]. In brief, mice were intraperitoneally injected with 2–3 mL of 4% thioglycolate medium (Eiken, Tokyo, Japan). After 72–96 h, PECs were harvested by peritoneal lavage with phosphate‐buffered saline (PBS), resuspended in RPMI 1640 medium (Gibco, USA) supplemented with 10% (v/v) heat‐inactivated fetal bovine serum (FBS; Gibco, USA), and seeded at densities of 1 × 10^6^ cells/well in 12‐well (or 6‐well) plates or 2 × 10^5^ cells/well in 48‐well plates. Cells were incubated at 37°C in a humidified 5% CO_2_ atmosphere. After 2 h of incubation, nonadherent cells were removed by gentle washing, and adherent macrophages were used for subsequent experiments.

For infection assays, WT and *Gsdmd* ^*−/−*^ PECs were challenged with PmCQ2 at a MOI of 1 for 9 h. Extracellular bacteria were eliminated by adding 100 μg/mL ciprofloxacin (Solarbio, China) for an additional 15 h. After 24 h of incubation, supernatants and cells lysates were collected for analysis. In inhibitor studies, cells were pretreated for 1 h with necrosulfonamide (NSA; 2 μM), disulfiram (10 μM), NAC (2 mM), MCC950 (10 μM), or Z‐VAD‐FMK (5 μM) prior to infection. To block potassium efflux, cells were pretreated with 5 mM KCl for 30 min before bacterial challenge.

### 2.4. *P. multocida* Infection *In Vivo*

WT and *Gsdmd* ^*−/−*^ mice were anesthetized via intraperitoneal (i.p.) injection of 1.25% tribromoethanol (LAT, Beijing, China) at a dosage of 0.2 mL/10 g body weight. Mice were intranasally inoculated with 1 × 10^3^ colony‐forming unit (CFU) of PmCQ2, while mock‐infected controls received an equal volume of sterile PBS, after which blood and lung tissues were aseptically collected. At 48 h postinfection, mice were euthanized by cervical dislocation, and blood and lung tissues were aseptically collected. Tissues were either homogenized in sterile PBS for bacterial load quantification or fixed in 4% paraformaldehyde (PFA) at room temperature for histological analysis.

### 2.5. Lactate Dehydrogenase (LDH) Cytotoxicity Assay

Cells were seeded in 96‐well plates and infected as described above. Following infection, host cell cytotoxicity was determined by measurement of the LDH release as reported [18]. Briefly, 50 µL of supernatant was mixed with 50 µL of reconstituted substrate mix and incubated for 30 min at room temperature in the dark. Reactions were terminated by adding 50 µL of stop solution, and the absorbance was measured at 490 nm or 492 nm using a microplate reader (Bio‐Rad, Japan).

### 2.6. Enzyme‐Linked Immunosorbent Assay (ELISA)

Following infection with PmCQ2, cell supernatants were collected and centrifuged (300× *g*, 10 min, 4°C) to remove debris. Cytokine secretion levels (IL‐1β, IL‐6, and TNF‐α) were quantified using ELISA kits (Thermo Fisher Scientific, Carlsbad, CA, USA; IL‐1β Cat. No. 88‐7013A‐88, IL‐6 Cat. No. 88‐7064‐88, TNF‐α Cat. No. 88‐7324‐88) in accordance with the manufacturer’s instructions.

### 2.7. Western Blotting

PECs were seeded in 12‐well plates and infected with PmCQ2 as described above. After 24 h of incubation at 37°C and 5% CO_2_, culture supernatant was collected and concentrated via precipitation with 20% (w/v) trichloroacetic acid (TCA) for 2 h at 4°C, followed by centrifugation at 14,000× *g* for 15 min. Cells were lysed in 1× SDS loading buffer (Beyotime, Beijing, China) and heated at 95°C for 5 min. Both concentrated supernatants and cell lysates were resolved by 10%–15% SDS‐polyacrylamide gel electrophoresis (SDS‐PAGE) and electrotransferred onto polyvinylidene difluoride (PVDF) membranes at 300 mA for 90 min. Membranes were blocked with 5% nonfat dry milk in tris‐buffered saline containing 0.1% Tween‐20 (TBST) for 1 h at room temperature. Primary antibodies including anti‐GSDMD (1:1000, Cell Signaling Technology, USA), anti‐caspase‐1 (1:1000, AdipoGen, San Diego, USA), anti‐caspase‐11 (1:1000, MedChemExpress, China), anti‐IL‐1β (1:500 Bioss, Beijing, China), and anti‐GAPDH (1:5000, Proteintech, China) were diluted in TBST with 2% bovine serum albumin (BSA) and incubated overnight at 4°C. Membranes were washed three times with TBST and incubated with HRP‐conjugated secondary antibodies (Beyotime, China; HRP‐labeled Goat Anti‐Rabbit IgG (H + L), 1:1000; HRP‐labeled Goat Anti‐Mouse IgG (H + L), 1:1000) for 1 h at room temperature. Protein bands were visualized using enhanced chemiluminescence (ECL) reagent (Biosharp, China) and imaged on automatic gel imaging system (Bio‐Rad, USA).

### 2.8. Confocal Microscopy

PECs were seeded into 35 mm glass‐bottom confocal dishes and infected with PmCQ2. At 24 h postinfection, cells were washed three times with PBS and then fixed with 4% PFA in PBS for 30 min at room temperature. Following fixation, cells were washed three times with PBS and blocked with 5% BSA for 1 h at room temperature. Subsequently, cells were incubated overnight at 4°C with a rabbit anti‐GSDMD primary antibody (1:100; ABclonal, China) diluted in blocking buffer. After 24 h, cells were washed three times with PBS and incubated with an anti‐rabbit fluorescent secondary antibody (1:500; Abcam, UK) for 1 h at room temperature in the dark. Following secondary antibody incubation, cell membranes were stained with wheat germ agglutinin (WGA) (Thermo Fisher Scientific, USA) dye for 30 min at 37°C, and nuclei were counterstained with DAPI for 5 min at room temperature, protected from light. After washing, slides were mounted with anti‐fade mounting medium, and images were captured using the CLSM610 laser‐scanning confocal microscope (SOPTOP, China) with consistent settings across all samples. Following infection, cells were harvested via trypsinization and fixed in 2.5% glutaraldehyde at 4°C overnight. Finally, samples were sent to the Lilai biomedicine experiment center (Sichuan, China) for transmission electron microscopy (TEM) analysis.

### 2.9. TEM

### 2.10. Propidium Iodide (PI) Staining

PECs were seeded in 12‐well plates and infected with PmCQ2 as previously described. At 24 h postinfection, cells were washed twice with PBS and stained using Hoechst/PI double staining kit (KeyGEN BioTECH, China, Cat. No. KGA1803‐100) according to the manufacturer’s instructions. After staining, cells were washed with PBS. Fluorescent images were acquired using an Olympus IX83 inverted microscope (Olympus, Japan) equipped with the appropriate fluorescent filters.

### 2.11. Detection of ROS

PECs were seeded in 12‐well plates and pretreated with NAC to modulate oxidative stress. Following pretreatment, cells were infected with PmCQ2 for 9 h. After infection, the culture medium was aspirated, and cells were washed three times with PBS buffer to thoroughly remove extracellular bacteria. To assess intracellular ROS levels, cells were incubated with 5 μmol/L dihydroethidium (DHE; Beyotime, China, S0063) in serum‐free medium for 30 min at 37°C, protected from light. After incubation, the cells were washed with PBS to remove unbound probe and replenished with serum‐free medium. Fluorescent images were acquired immediately using an Olympus IX83 inverted microscope (Olympus, Japan) equipped with the appropriate fluorescent filters.

### 2.12. siRNA Transfection

Cells were transfected with 50 nM caspase‐11‐targeting siRNA (si‐Casp11; sequence: 5^′^‐CCGUACACGAAAGGCUCUUAUTT‐3^′^) or scrambled control siRNA (si‐Control; sequence: 5^′^‐AUAAGAGCCUUUCGUGUACGGTT‐3^′^) (Genecreate Biological, China) using Lipofectamine 3000 (Thermo Fisher Scientific, USA). Transfection complexes were prepared at a 1:1 ratio (siRNA: Lipofectamine 3000) and incubated with cells for 36 h. Following transfection, cells were infected with PmCQ2 as previously described. Supernatants and lysates were collected 24 h postinfection for analysis of pyroptosis markers via LDH release assay, IL‐1β ELISA, immunoblotting, and real‐time (RT)‐PCR analysis.

### 2.13. Quantitative RT‐PCR

PECs were infected with PmCQ2 as described. Total RNA was extracted using TRIzol Reagent (Accurate Biology, China, Cat. No. AG11728) and reverse‐transcribed into cDNA using the PrimeScript RT Reagent Kit (TaKaRa, Japan) with oligo (dT) primers. Quantitative RT‐PCR was performed on a Bio‐Rad CFX96 system (Bio‐Rad, USA) using SYBR Green Premix Ex Taq. Primer sequences were as follows: β‐actin forward, 5^’^‐GTGACGTTGACATCCGTAAAGA‐3^’^ and reverse, 5^′^‐GCCGGACTCATCGTACTCC‐3^′^; caspase‐11 forward, 5^′^‐ ACAAACACCCTGACAAACCAC‐3^′^ and reverse, 5^′^‐ CACTGCGTTCAGCATTGTTAAA‐3^′^. Reactions 20 μL contained 10 μL SYBR Green mix, 0.5 μM primers, and 2 μL cDNA. Thermal cycling conditions were 95°C, 2 min; 95°C, 5 s, 40 cycles; 60°C, 30 s; 95°C, 5 s; 60°C, 5 s; and 95°C, 2 min. Relative mRNA levels were normalized against the expression levels of β‐actin.

### 2.14. Adhesion and Invasion Assays

PECs were seeded and processed as described. Adherent macrophages were challenged with PmCQ2 at MOI of 1 for 12 h at 37°C under 5% CO_2_. Following infection, nonadherent bacteria were removed by three washes with sterile PBS. Cells were lysed with 0.1% Triton X‐100 (Sigma–Aldrich, USA) in PBS for 10 min at room temperature. Lysates were serially diluted in PBS, plated on Martin agar (Solarbio, China) supplemented with 10% heat‐inactivated horse serum (Evergreen Biological, China), and incubated aerobically at 37°C for 12–18 h. Adherent bacteria were quantified by enumerating CFUs. For invasion assays, after the 12 h infection, extracellular bacteria were eliminated by incubating cells with 100 μg/mL ciprofloxacin (Solarbio, China) in RPMI 1640 medium for 30 min at 37°C. Cells were washed three times with PBS, lysed with 0.1% Triton X‐100, and processed as above. Intracellular bacteria were quantified by CFU counts.

### 2.15. Histopathology

Following intranasal inoculation with PmCQ2, mice were euthanized at 48 h postinfection, and the dissected tissues were fixed in 4% PFA; Sigma–Aldrich, USA for 24 h at 4°C. Finally, samples were sent to the Lilai biomedicine experiment center (Sichuan, China) for H&E staining analysis.

### 2.16. Statistical Analysis

Data are presented as mean ± SEM of three independent experiments, each containing triplicate biological replicates (*n* = 3 per group). One‐way ANOVA was used to analyze the statistical significance among different groups. All analyses were performed using GraphPad Prism software (San Diego, CA, USA). Statistical significance is shown as  ^∗^ *p* ≤ 0.05,  ^∗∗^ *p* ≤ 0.01,  ^∗∗∗^ *p* ≤ 0.001, and ns = not significant.

## 3. Results

### 3.1. *P. multocida* Infection Induces Macrophage Pyroptosis

To investigate whether *P. multocida* induces pyroptosis, we first assessed cellular morphology by optical microscopy in macrophages. PmCQ2 infection elicited substantial macrophage cell death, marked by cytoplasmic swelling and membrane blebbing (Figure 1A). Subsequent LDH release assays demonstrated a time‐dependent increase in extracellular LDH levels (2 h, 6 h, 12 h, 24 h), indicative of progressive plasma membrane disruption (Figure 1B). To interrogate the molecular mechanism underlying this cell death, we performed Western blot analysis. Significant cleavage of pyroptosis‐associated proteins (GSDMD, caspase‐1, and caspase‐11) was observed during infection (Figure 1C), consistent with activation of the canonical and noncanonical pyroptotic pathways. TEM further confirmed hallmark pyroptotic morphology, including plasma membrane rupture, cytoplasmic swelling with organelle disintegration, and mitochondrial contraction (Figure 1D). Collectively, these data demonstrate that *P. multocida* infection triggers pyroptosis in macrophages through activation of GSDMD and caspases, culminating in lytic cell death.

Figure 1
*P. multocida* infection induces macrophage pyroptosis. Peritoneal macrophages from WT (C57BL/6) mice were infected with PmCQ2 at an MOI of 1 for the indicated times of 0 h, 2 h, 6 h, 12 h, and 24 h. The morphology of primary peritoneal macrophages infected with PmCQ2 was observed by light microscopy (A). The level of cell death was quantitatively assessed by lactate dehydrogenase (LDH) release assay (B). Western blot was used to measure the protein levels of GSDMD‐N, caspase‐1, and caspase‐11 (C). The destruction of cell membrane integrity and the characteristic morphological changes of pyroptosis were observed by transmission electron microscopy (TEM) (D). Data are represented as mean ± SEM of three independent experiments of triplicate samples per experiment.  ^∗^ *p* ≤ 0.05,  ^∗∗^ *p* ≤ 0.01,  ^∗∗∗^ *p* ≤ 0.001.

**Figure:**
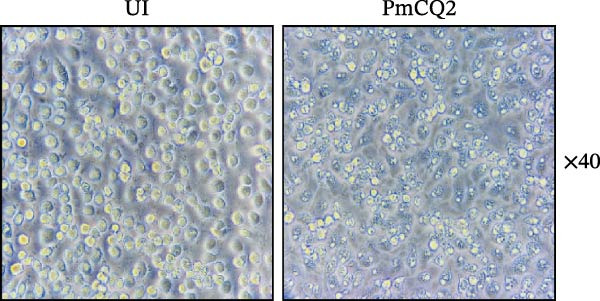
(A)

**Figure:**
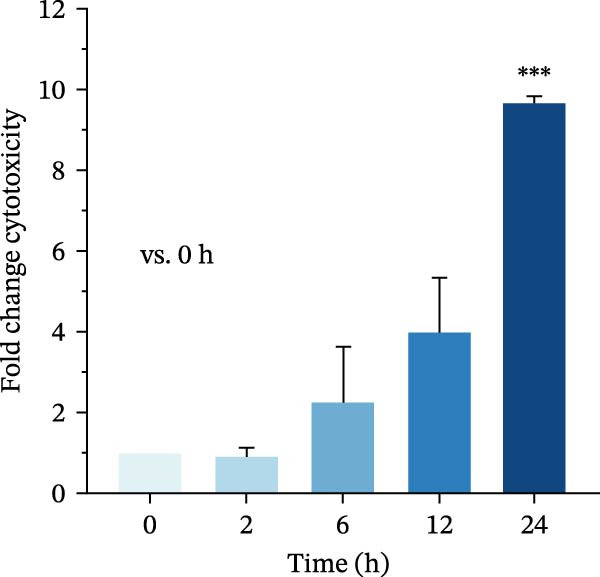
(B)

**Figure:**
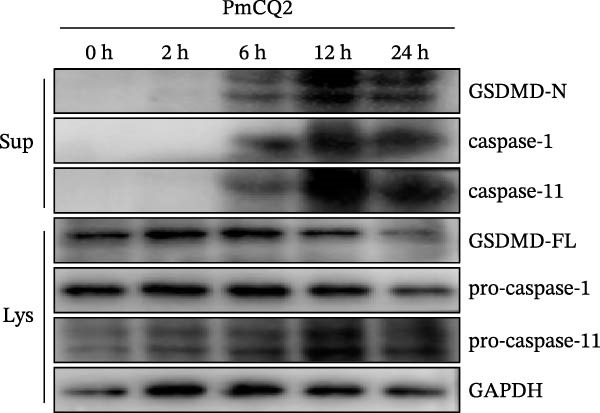
(C)

**Figure:**
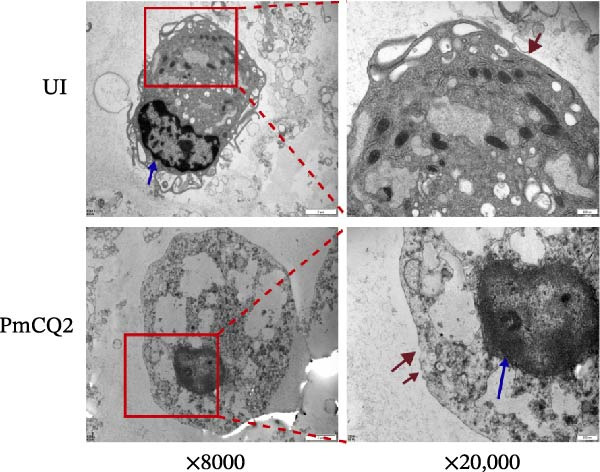
(D)

### 3.2. *P. multocida* Induces Pyroptosis via GSDMD

To further define the role of GSDMD in *P. multocida*‐induced pyroptosis, we utilized the pharmacological inhibitor NSA, which blocks GSDMD oligomerization. NSA treatment markedly attenuated PmCQ2‐induced cytotoxicity in macrophages (Figure 2A) and IL‐1β secretion (Figure 2B). Western blot analysis corroborated these findings, showing that NSA suppressed GSDMD cleavage and IL‐1β maturation (Figure 2C). To further validate these results, we infected PECs from *Gsdmd* ^*−/−*^ mice with PmCQ2. Genetic ablation of GSDMD abolished pyroptotic cell death (Figure 2D) and IL‐1β release (Figure 2E). In contrast, levels of TNF‐α and IL‐6 remained unaffected by GSDMD (Figure 2F, G), indicating specificity of GSDMD in regulating IL‐1β release. Additionally, disulfiram, an FDA‐approved GSDMD inhibitor, was utilized to further assess GSDMD’s involvement in pyroptosis. Confocal microscopy analysis revealed that pretreatment with disulfiram significantly attenuated membrane damage in infected macrophages (Figure 2H and Supporting Information 1: Figure S1). These findings demonstrate that GSDMD plays a crucial role in *P. multocida*‐induced IL‐1β secretion and pyroptosis.

Figure 2
*P. multocida* induces pyroptosis via GSDMD. PECs from WT (C57BL/6) or *Gsdmd* ^*−/−*^ (C57BL/6) mice were uninfected or infected with PmCQ2 (MOI = 1) for 24 h. PECs were pretreated with NSA (2 μM) for 1 h prior to infection. Following infection, cell death was measured by lactate dehydrogenase (LDH) release (A). In addition, the secretion level of the inflammatory factor IL‐1β was determined by ELISA (B) and the activation level of the protein GSDMD‐N and IL‐1β was determined by Western blot (C). PI uptake of PECs from WT and *Gsdmd* ^*−/−*^ mice was detected (D). ELISA measured the secretion levels of IL‐1β, TNF‐α, and IL‐6 in PECs from WT and *Gsdmd* ^*−/−*^ mice (E‐G). Representative confocal microscopy images of PECs stained with wheat germ agglutinin (WGA, green) to label cell membranes. Panels show: (H1) Untreated cells (UI), (H2) Disulfiram‐treated, (H3) PmCQ2‐infected, and (H4) PmCQ2 infection following disulfiram pretreatment (H). Data are represented as mean ± SEM of three independent experiments of triplicate samples per experiment.  ^∗^ *p* ≤ 0.05,  ^∗∗^ *p* ≤ 0.01,  ^∗∗∗^ *p* ≤ 0.001.

**Figure:**
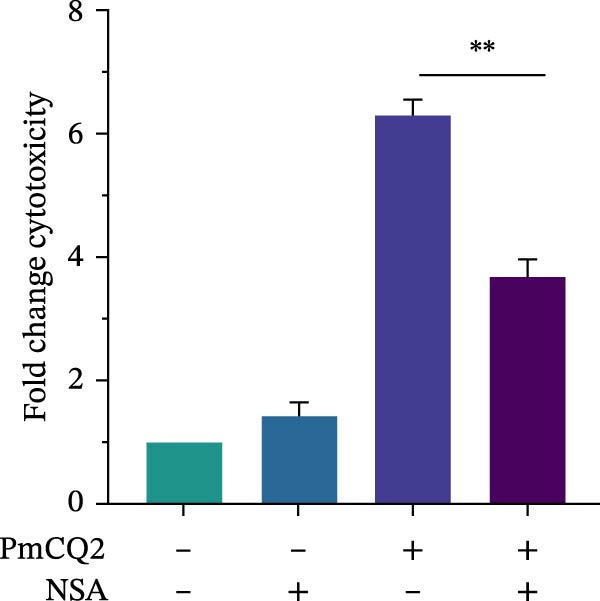
(A)

**Figure:**
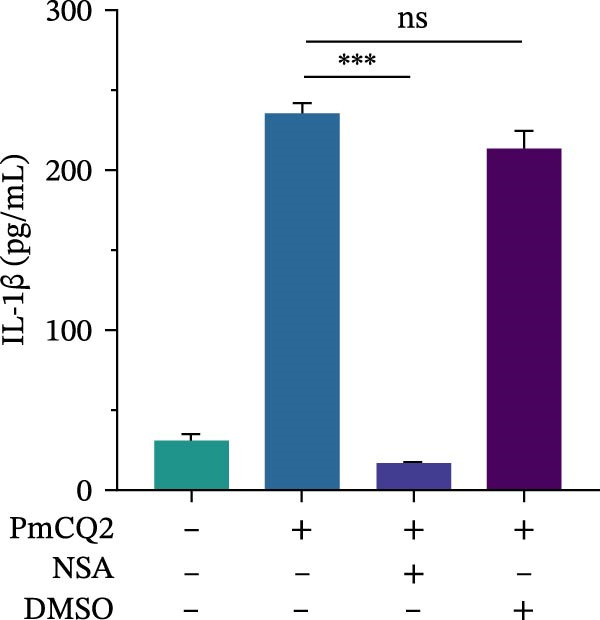
(B)

**Figure:**
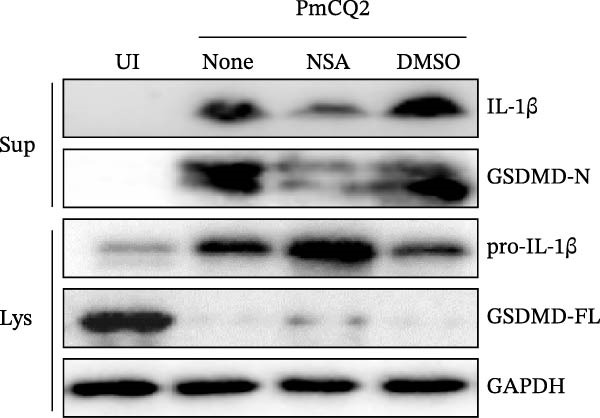
(C)

**Figure:**
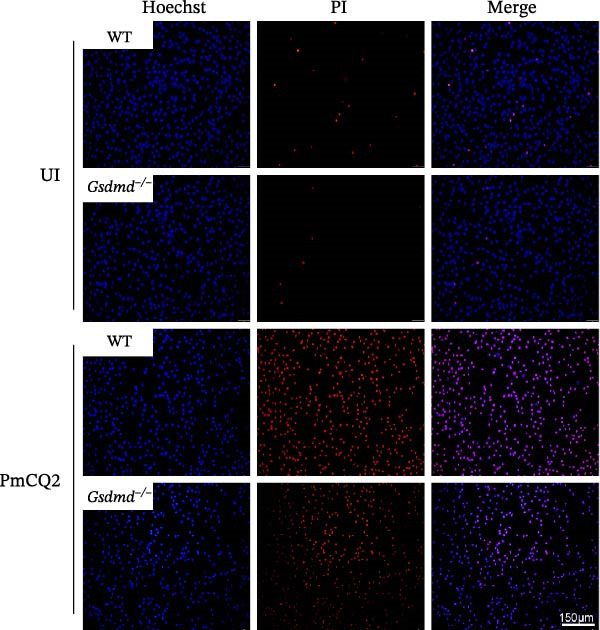
(D)

**Figure:**
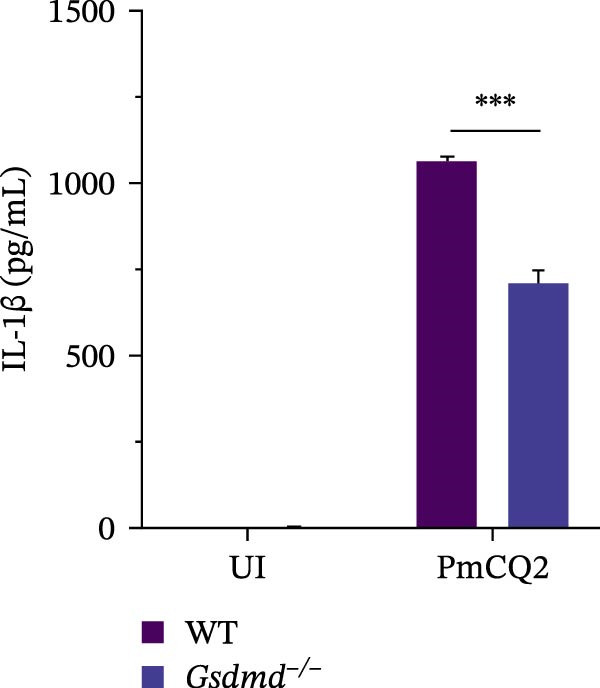
(E)

**Figure:**
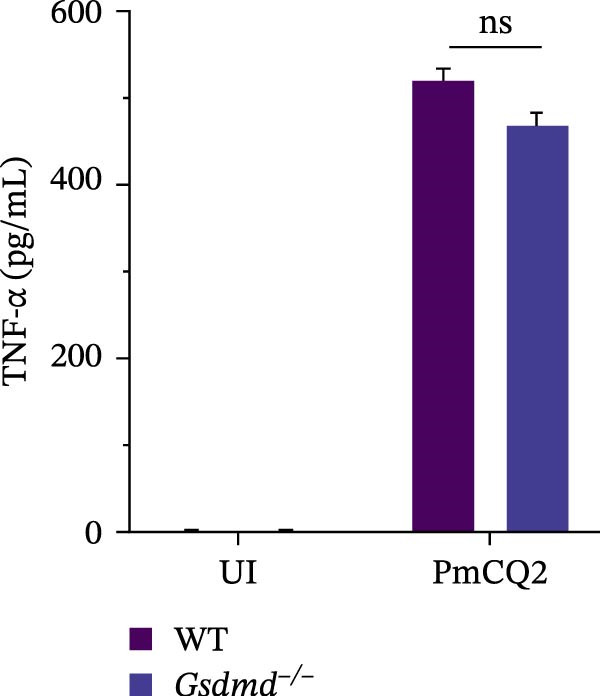
(F)

**Figure:**
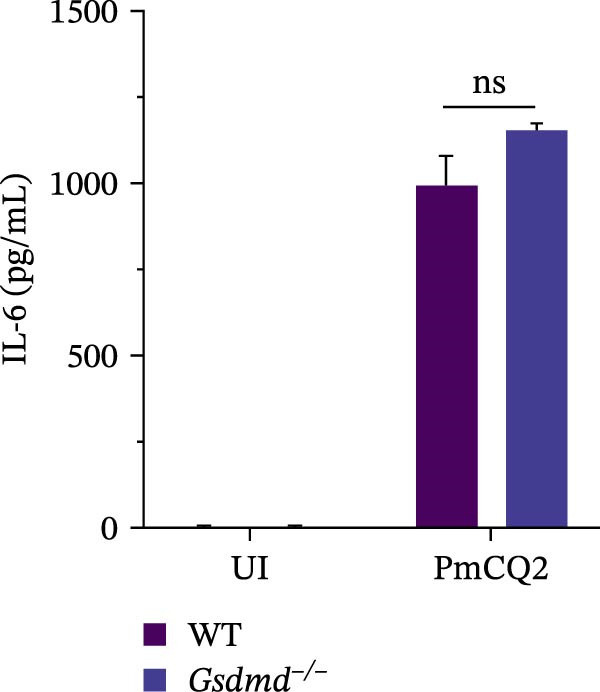
(G)

**Figure:**
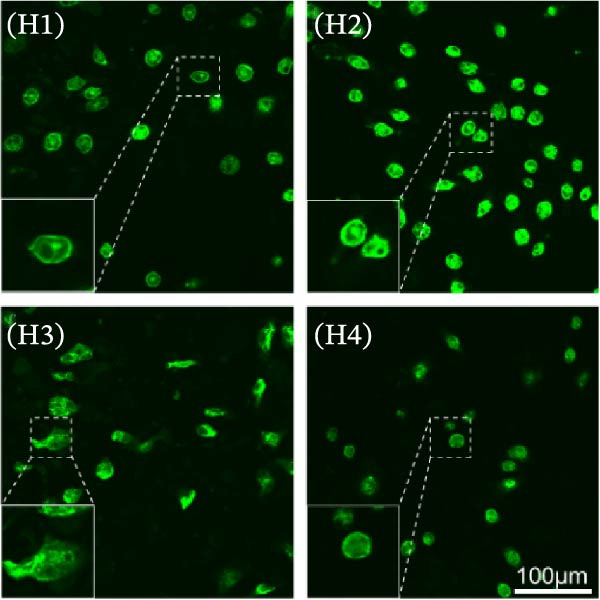
(H)

### 3.3. *P. multocida* Activates GSDMD to Mediate Pyroptosis Through Activation of NLRP3 Inflammasome

The activation of the NLRP3 inflammasome is the core link of pyroptosis. To evaluate the involvement of NLRP3 in *P. multocida*‐induced pyroptosis, primary macrophages were pretreated with the NLRP3‐specific inhibitor MCC950 prior to infection. MCC950 significantly attenuated cleavage of GSDMD into its active N‐terminal fragment (GSDMD‐N) and reduced caspase‐1 activation (Figure 3A). Concurrently, MCC950 suppressed IL‐1β secretion (Figure 3B) and diminished pyroptosis‐associated cytotoxicity (Figure 3C), indicating NLRP3 dependence in this pathway.

Figure 3
*P. multocida* activates GSDMD to mediate pyroptosis through activation of NLRP3 inflammasome. PECs were pretreated with MCC950 (10 μM) for 30 min prior to infection. After infection, Western blot was used to detect the protein levels of GSDMD‐N and caspase‐1 (A), ELISA was used to determine the secretion of IL‐1β (B), and macrophage death was measured by the LDH release (C). Furthermore, PECs were pretreated with KCl (5 mM) for 30 min prior to infection. After infection, cell death was measured by LDH release (D) and PI uptake (E) in macrophages. The protein levels of GSDMD‐N and caspase‐1 (F) and the secretion of IL−1β were determined (G). Data are represented as mean ± SEM of three independent experiments of triplicate samples per experiment.  ^∗^ *p* ≤ 0.05,  ^∗∗^ *p* ≤ 0.01.

**Figure:**
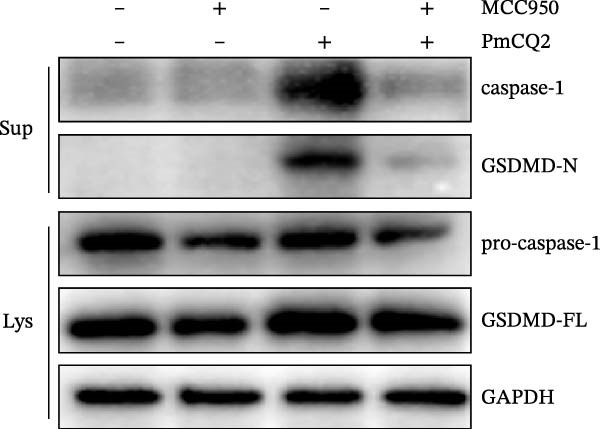
(A)

**Figure:**
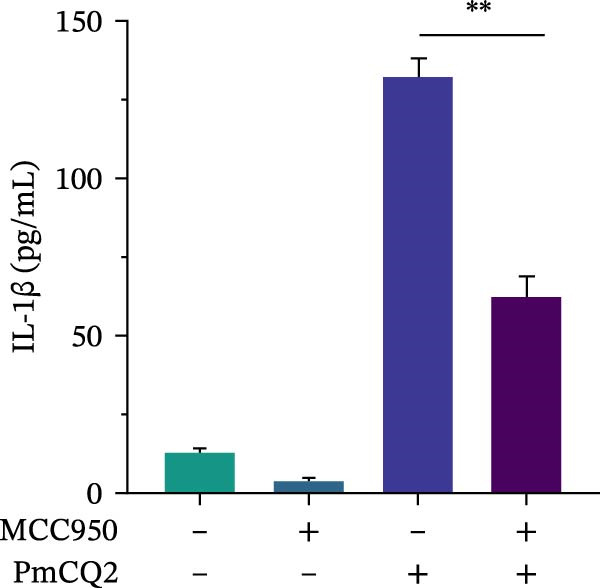
(B)

**Figure:**
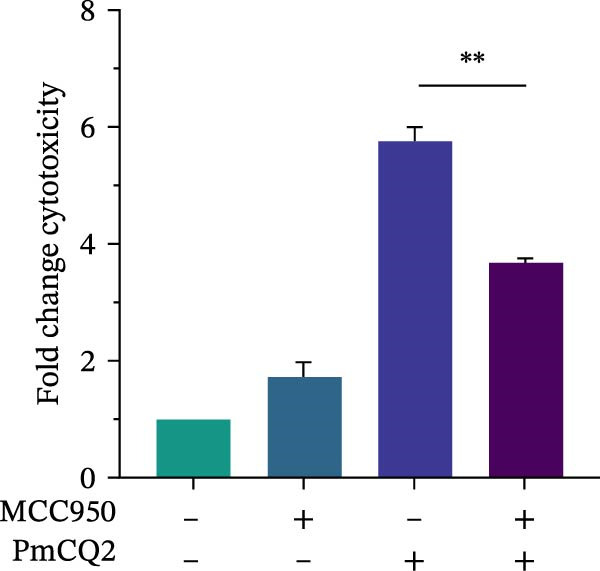
(C)

**Figure:**
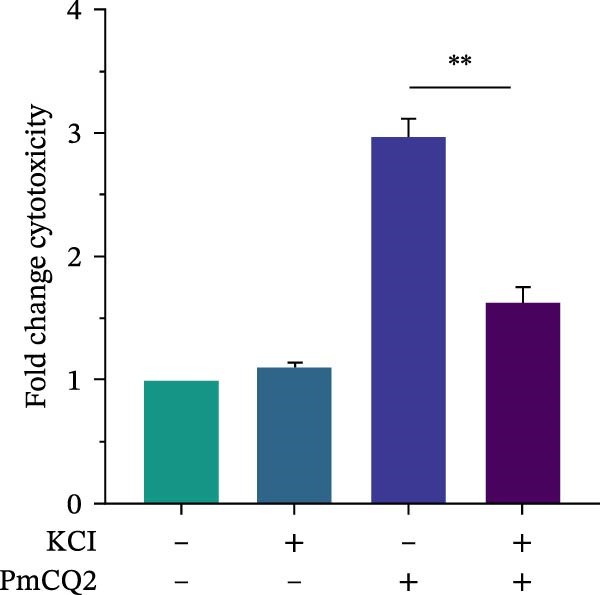
(D)

**Figure:**
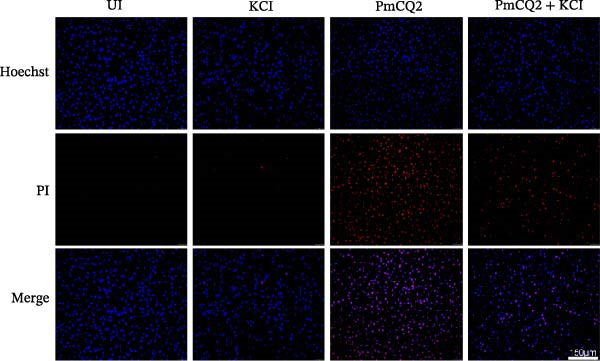
(E)

**Figure:**
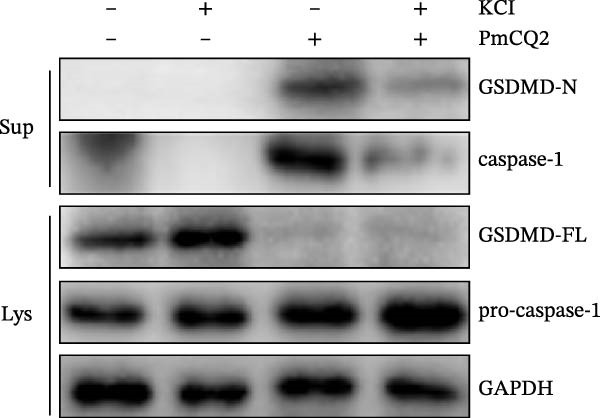
(F)

**Figure:**
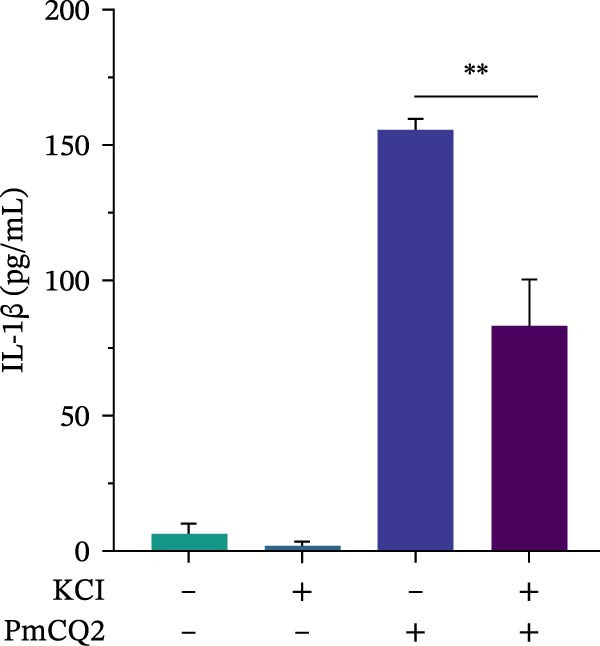
(G)

Intracellular K^+^ efflux is a known trigger for NLRP3 inflammasome assembly, enabling NLRP3‐NEK7 interaction and subsequent activation [19]. To assess the role of K^+^ efflux in *P. multocida*‐induced pyroptosis, extracellular KCl was used to stabilize intracellular K^+^ levels. KCl pretreatment substantially reduced pyroptotic cell death (Figure 3D, E), suppressed GSDMD‐N generation and caspase‐1 activation (Figure 3F), and attenuated IL‐1β release (Figure 3G), indicating that K^+^ efflux is essential for inflammasome activation and subsequent pyroptosis during *P. multocida* infection. ROS accumulation is recognized as a key upstream signal for NLRP3 inflammasome activation. To investigate the role of ROS in PmCQ2‐induced pyroptosis, cells were pretreated with NAC, broad‐spectrum ROS inhibitor. NAC treatment significantly reduced cell death induced by PmCQ2 infection (Supporting Information 2: Figure S2A). Western blot analysis further demonstrated that NAC effectively inhibited the generation of GSDMD‐N and the activation of caspase‐1 (Supporting Information 2: Figure S2B). Additionally, ROS‐sensitive DHE fluorescence imaging revealed that NAC pretreatment markedly suppressed intracellular ROS production (Supporting Information 2: Figure S2C), suggesting that the ROS signaling pathway plays a critical role in PmCQ2‐induced NLRP3 inflammasome activation and pyroptosis. Collectively, these results demonstrate that K^+^ efflux and ROS generation act as critical upstream signals essential for NLRP3 inflammasome activation and pyroptosis during *P. multocida* infection.

### 3.4. *P. multocida* Induces Pyroptosis by Activating GSDMD Through the Noncanonical Pathway Caspase‐11

While canonical NLRP3 inflammasome signaling dominates pyroptosis research, emerging evidence suggests that certain Gram‐negative bacteria (e.g., *Shigella flexneri*, *E. coli*, and *Salmonella typhimurium*) directly activate caspase‐11 via the noncanonical pathway, leading to GSDMD cleavage and pyroptosis [20, 21]. To determine whether *P. multocida* similarly engages caspase‐11, we performed siRNA‐mediated knockdown of caspase‐11 in macrophages. Efficient silencing was confirmed by reduced caspase‐11 protein and mRNA levels (Figure 4A, B). Caspase‐11 knockdown markedly attenuated GSDMD cleavage (evidenced by diminished GSDMD‐N levels; Figure 4A) and suppressed LDH release (Figure 4C). Furthermore, pretreatment with the pan‐caspase inhibitor Z‐VAD‐FMK abolished both GSDMD cleavage and IL‐1β maturation during infection (Figure 4D,E), confirming that caspase activity is essential for *P. multocida*‐triggered pyroptosis. These results demonstrate that caspase‐11 is essential for *P. multocida*‐induced GSDMD activation and pyroptosis, implicating the noncanonical pathway in this process.

Figure 4
*P. multocida* induces pyroptosis by activating GSDMD through the noncanonical pathway caspase‐11. PECs were transfected with siRNA for 48 h to knockdown caspase‐11. After transfection, Western blot and qPCR were used to detect GSDMD‐N and caspase‐11 protein expression (A) and caspase‐11 mRNA expression (B). Macrophage death was measured by the LDH release (C). In addition, PECs were pretreated with Z‐VAD‐FMK (5 μM) for 30 min prior to infection. After infection, the protein levels of GSDMD‐N, caspase‐1, and caspase‐11 (D) and the secretion of IL−1β were determined (E). Data are represented as mean ± SEM of three independent experiments of triplicate samples per experiment.  ^∗^ *p* ≤ 0.05,  ^∗∗^ *p* ≤ 0.01,  ^∗∗∗^ *p* ≤ 0.001.

**Figure:**
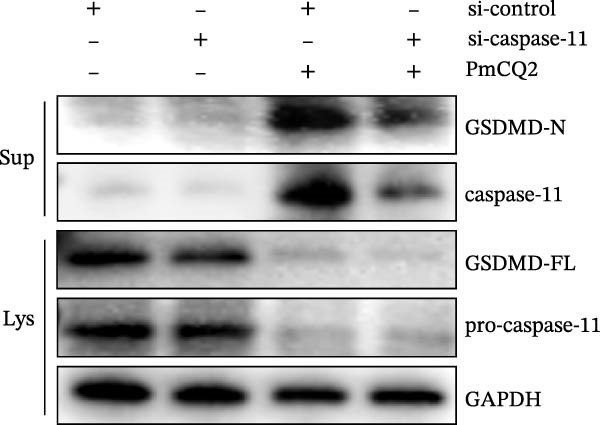
(A)

**Figure:**
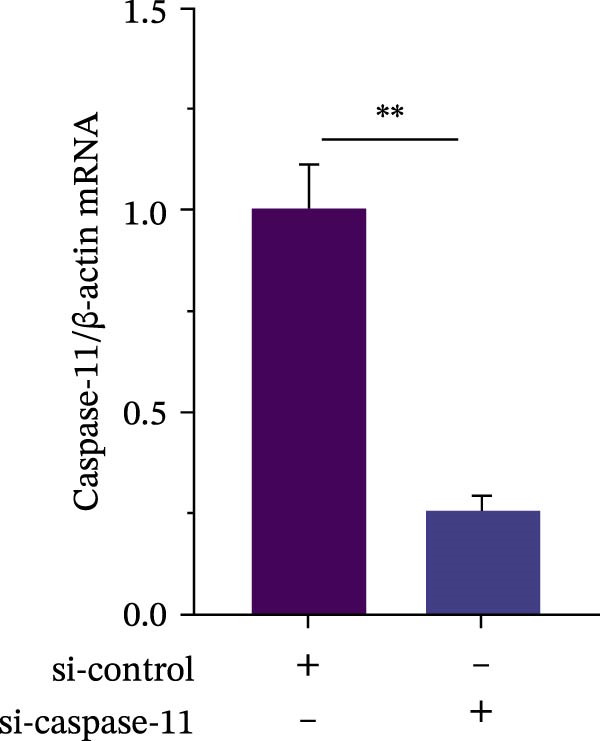
(B)

**Figure:**
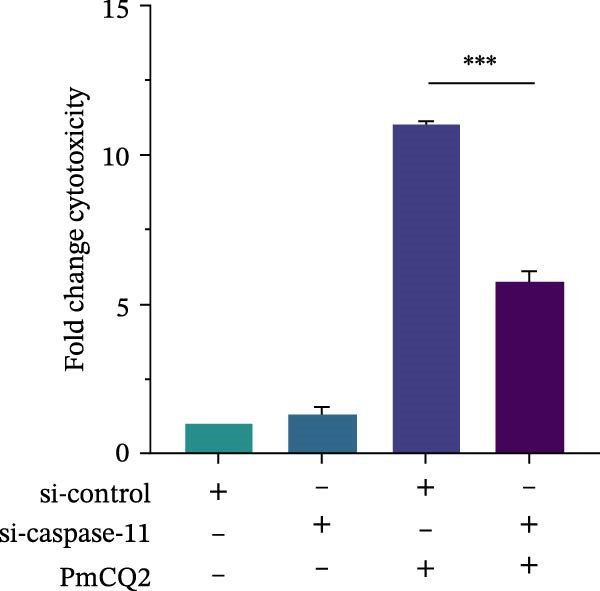
(C)

**Figure:**
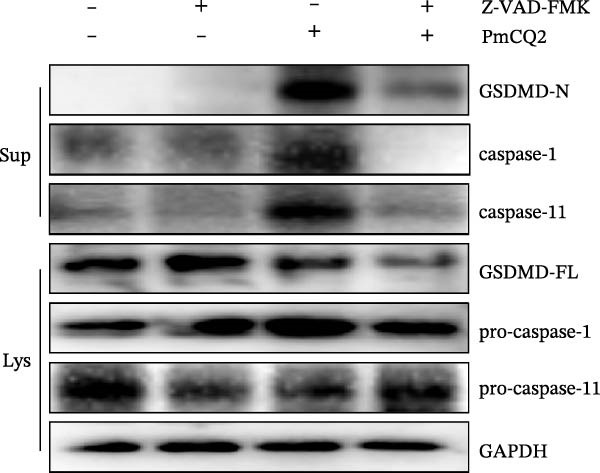
(D)

**Figure:**
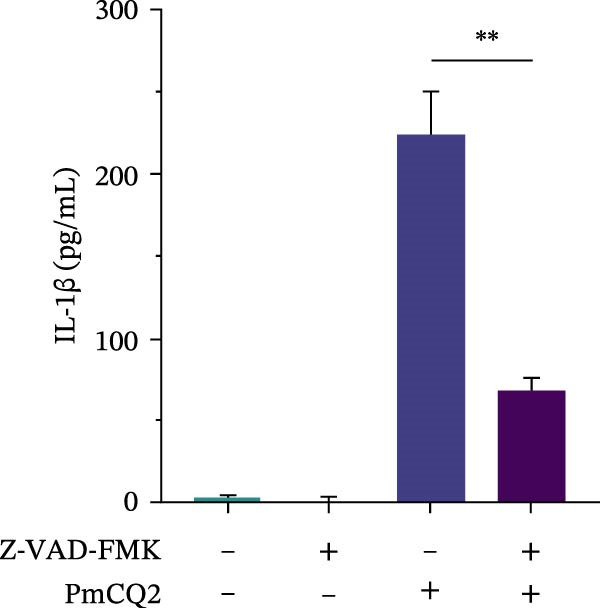
(E)

### 3.5. GSDMD Contributes to Bacterial Invasion and Excessive Immunopathology

To elucidate the role of GSDMD in host defense against *P. multocida*, we first assessed bacterial adhesion and invasion in macrophages pretreated with the GSDMD oligomerization inhibitor NSA. NSA treatment dose‐dependently reduced bacterial adhesion and invasiveness (Figure 5A, B), suggesting that GSDMD activity promotes bacterial entry into macrophages. We further evaluated host–pathogen interactions using WT and *Gsdmd* ^*−/−*^ mice intranasally inoculated with PmCQ2 (1 × 10^3^ CFU). IL‐1β levels in lung tissues were significantly elevated in WT mice compared to *Gsdmd* ^*−/−*^ mice (Figure 5C), consistent with GSDMD‐dependent inflammasome activation. However, *Gsdmd* ^*−/−*^ mice exhibited higher bacterial loads in lungs and blood (Figure 5D, E), indicating impaired pathogen clearance. Paradoxically, despite enhanced bacterial colonization, *Gsdmd* ^*−/−*^ mice showed reduced pulmonary inflammation, with diminished leukocyte infiltration observed histologically (Figure 5F), and improved survival (Figure 5G). These findings suggest a dual role for GSDMD: it promotes IL‐1β‐driven antimicrobial defense, limiting bacterial dissemination in WT mice, while its absence in *Gsdmd* ^*−/−*^ mice attenuates immunopathology at the cost of compromised pathogen control. This dichotomy underscores GSDMD’s critical function in balancing inflammatory responses and bacterial containment during *P. multocida* infection.

Figure 5GSDMD contributes to bacterial invasion and excessive immunopathology. PECs were pretreated with different concentrations of NSA (0.5 µM, 1 µM, and 2 µM) for 1 h prior to infection. The number of adherent and phagocytic PmCQ2 in cells (A and B). WT (*n* ≥ 5) and *Gsdmd* ^*−/−*^ (*n* ≥ 5) mice were infected intranasally with PmCQ2 (1000 CFU), and sterilized PBS was used as a control. At 48 h post‐nfection (hpi), blood and lung tissue were obtained. ELISA was used to detect the secretion of IL‐1β in lung tissue (C). Bacterial load in lungs and blood (D and E). H&E staining of lungs tissue (F). Survival rates of mice (G). Data are represented as mean ± SEM of three independent experiments of triplicate samples per experiment.  ^∗^ *p* ≤ 0.05,  ^∗∗^ *p* ≤ 0.01,  ^∗∗∗^ *p* ≤ 0.001.

**Figure:**
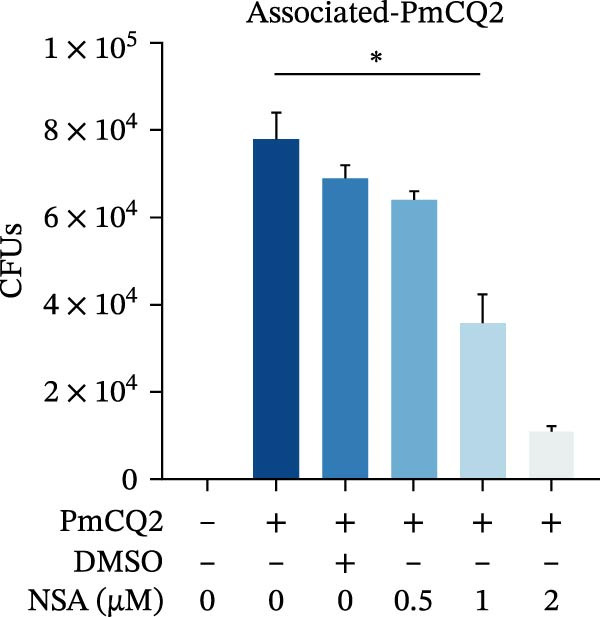
(A)

**Figure:**
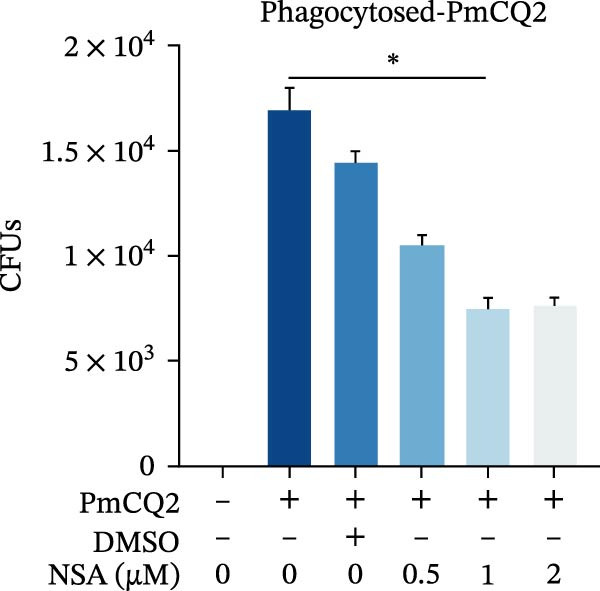
(B)

**Figure:**
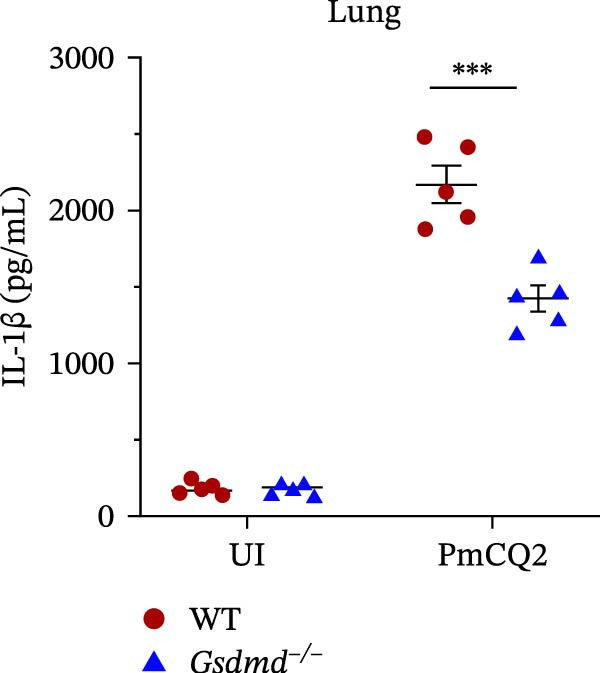
(C)

**Figure:**
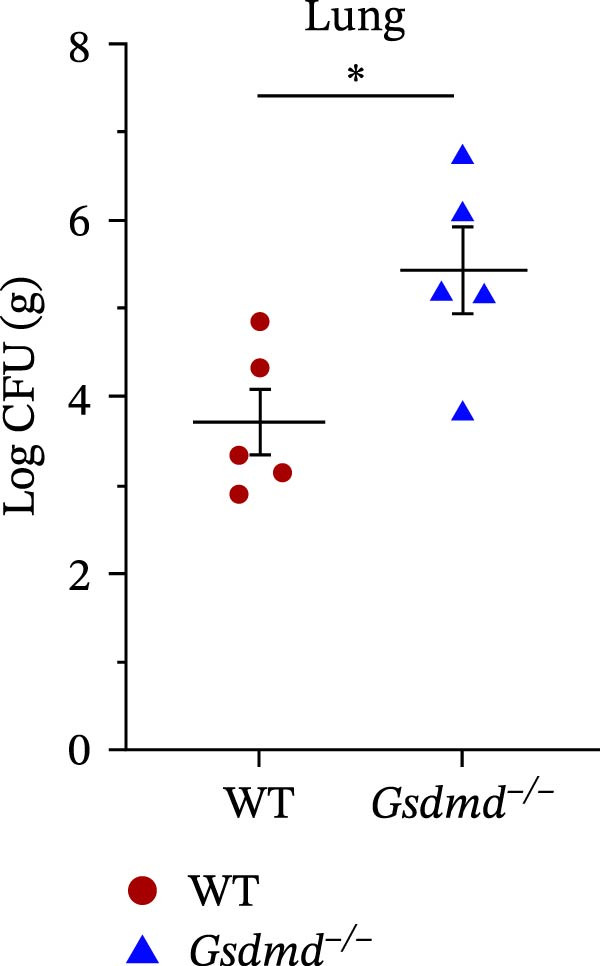
(D)

**Figure:**
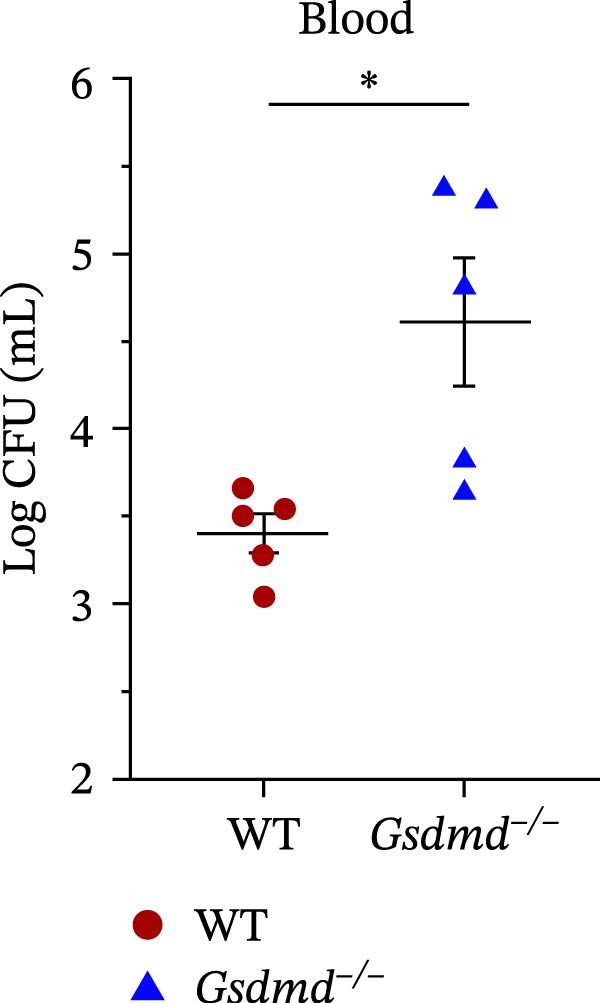
(E)

**Figure:**
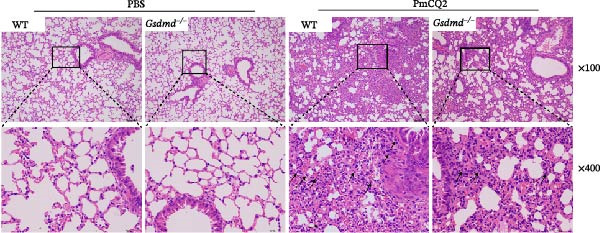
(F)

**Figure:**
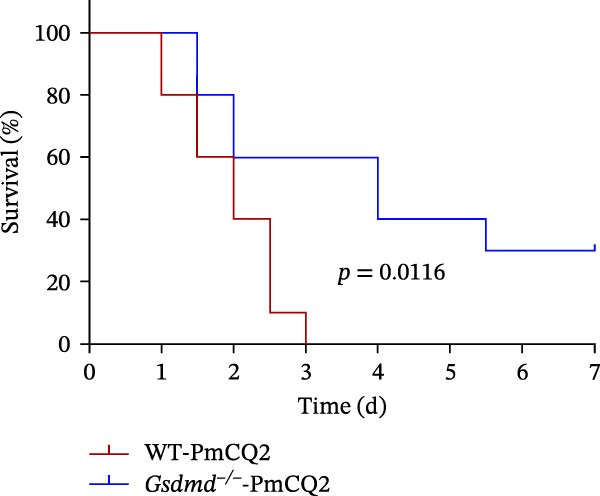
(G)

## 4. Discussion

**Figure 6:**
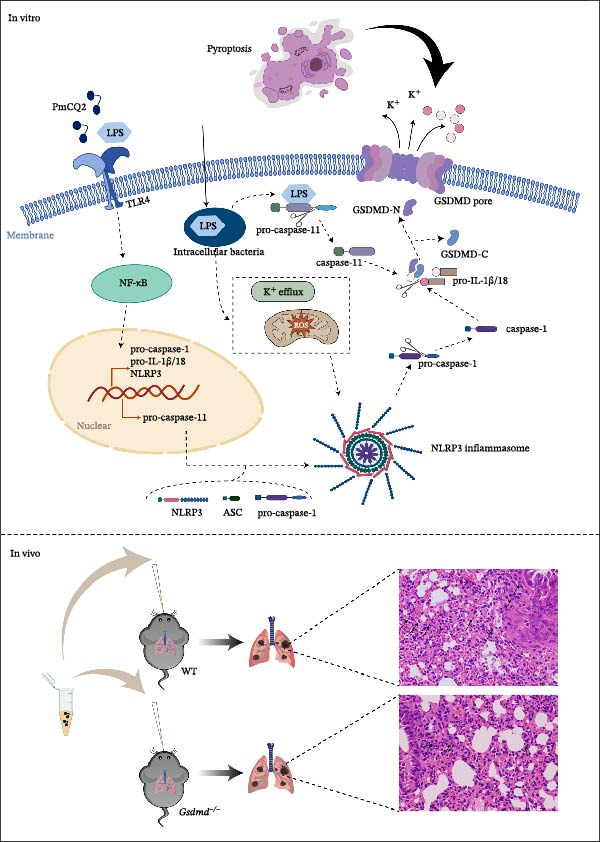
PmCQ2 induces pyroptosis via both canonical and noncanonical inflammasome pathways in vitro and GSDMD deficiency alleviates lung pathology in vivo.

The pathogenesis of PmCQ2 hinges on its ability to manipulate host immune responses, particularly through the activation of the NLRP3 inflammasome and GSDMD‐mediated pyroptosis, as demonstrated in the current study [22]. Our findings reveal that PmCQ2 engages both canonical and noncanonical inflammasome pathways to drive GSDMD‐mediated pyroptosis in macrophages, with potassium efflux–dependent NLRP3 activation and caspase‐11 sensing of cytosolic LPS converging on GSDMD cleavage to promote IL‐1β release and cell lysis—all of which can both restrict bacterial replication and, when unchecked, exacerbate host immunopathology. Inhibition of NLRP3 with MCC950 or extracellular KCl blockade of K^+^ efflux markedly reduces caspase‐1 activation, GSDMD‐N generation, LDH release, and IL‐1β secretion, confirming the primacy of ionic perturbations in inflammasome assembly during *P. multocida* infection. Genetic ablation or pharmacologic blockade of GSDMD abrogates pyroptotic death and cytokine release, underscoring GSDMD’s role as the terminal executioner pore‐former in both canonical and noncanonical pathways (Figure 6). These findings align with broader trends in microbial pathogenesis research, where pyroptosis has emerged as a double‐edged sword in host–pathogen interactions—essential for pathogen clearance but detrimental when dysregulated, leading to tissue damage and systemic inflammation.

Our data reveal that PmCQ2 infection triggers GSDMD‐dependent pyroptosis in macrophages via both canonical (NLRP3/caspase‐1) and noncanonical (caspase‐11) pathways, a mechanism consistent with observations in other bacterial infections, such as *Salmonella* and *Yersinia* [23, 24]. Silencing caspase‐11 or pan‐caspase inhibition virtually abolishes GSDMD cleavage and LDH release, demonstrating that cytosolic LPS sensing by noncanonical caspase‐11 is indispensable for *P. multocida*‐induced pyroptosis alongside caspase‐1 signaling. This dual engagement mirrors recent studies showing that intracellular LPS directly activates caspase‐11 (or human caspase‐4/5), which then cleaves GSDMD and amplifies inflammation through pannexin‐1–mediated ATP release and P2X7 receptor signaling [25]. Such convergence not only ensures robust antimicrobial responses but also poses the risk of collateral tissue damage if regulatory checkpoints fail. The dual activation pathways suggest evolutionary redundancy in PmCQ2’s strategy to ensure robust inflammatory responses, which may facilitate bacterial dissemination by destabilizing host barriers. The apparent discrepancy between our in vitro and in vivo bacterial burdens reflects the complexity of host defenses. Although inhibiting GSDMD reduced bacterial entry into macrophages in vitro, effective bacterial control in vivo relies on coordinated immune responses. GSDMD‐dependent IL‐1β release is a critical driver of neutrophil recruitment [7]; therefore, its absence in *Gsdmd* ^*−/−*^ mice likely compromises systemic clearance, leading to increased bacterial loads. Furthermore, the GSDMD N‐terminal fragment has been shown to exert direct bactericidal activity by binding bacterial membrane lipids [26]. The loss of this mechanism may further contribute to the elevated bacterial burden in knockout mice. Paradoxically, despite higher bacterial loads, *Gsdmd* ^*−/−*^ mice exhibited attenuated lung injury and improved survival. This highlights the pathogenic consequences of excessive lytic pyroptosis. GSDMD‐mediated membrane rupture releases inflammatory mediators that can precipitate lethal immunopathology [20]. In the absence of GSDMD, this lytic inflammatory burst is blunted, shifting the host response from pathogen resistance toward disease tolerance [27]. Collectively, these findings indicate that while GSDMD helps limit bacterial growth, its excessive activation imposes a severe cost through inflammation‐driven tissue injury, which ultimately outweighs its protective antibacterial benefits.

A critical insight from this work is the therapeutic potential of targeting GSDMD. We observed that pharmacological inhibition of GSDMD with NSA significantly reduced not only pyroptotic cell death and inflammatory signals but also bacterial adhesion and invasion in macrophages. A plausible mechanistic explanation is that GSDMD pore formation alters plasma membrane architecture, including membrane permeability and surface dynamics, which may facilitate bacterial attachment and entry. By preventing GSDMD oligomerization and pore formation, NSA likely preserves membrane integrity and limits these infection‐permissive membrane changes, thereby reducing bacterial adhesion and invasion [28]. Although this effect is indirect, it is consistent with the known role of GSDMD‐mediated membrane remodeling during pyroptosis. This approach aligns with recent advances in sepsis management, where metabolic interventions—like time‐restricted feeding (TRF)—reshape gut microbiota and metabolites (e.g., 3‐hydroxybutyrate) to suppress ferroptosis and inflammation [29]. Such strategies could complement traditional antibiotics, especially given *P. multocida*’s alarming resistance rates to tetracyclines (79.2%−81.3%) [30]. For instance, combining NLRP3 inhibitors with pathogen‐specific therapies, as seen in *Pseudomonas putida*‐nanoparticle synergies for plant disease control [31], might enhance efficacy while reducing collateral tissue damage.

The study also raises questions about the specificity of *P. multocida* virulence factors driving pyroptosis. While capsular polysaccharides and LPS are implicated, parallels to *Macrorhabdus ornithogaster* infections—where fungal toxins disrupt host nutrient absorption—hint at potential toxin‐mediated mechanisms [32]. Although cytosolic LPS is the primary trigger of caspase‐11 activation, additional *P. multocida* virulence‐associated mechanisms may facilitate LPS access to the host cytosol. For instance, Gram‐negative bacteria are known to secrete outer membrane vesicles (OMVs), which can function as delivery vehicles for LPS and promote its cytosolic localization [33]. Additionally, bacterial toxins that perturb phagosomal or endosomal membrane integrity may enable leakage of LPS into the cytoplasm [34, 35]. Thus, while LPS likely represents the proximal activator of caspase‐11 in this context, other bacterial components may indirectly contribute by enhancing cytosolic LPS delivery and amplifying noncanonical inflammasome activation. Further investigation into *P. multocida*’s exotoxins, akin to D‐type strains causing porcine atrophic rhinitis through dermonecrotic toxins [36], could elucidate molecular triggers of inflammasome activation. In addition to GSDMD, the potential involvement of other gasdermin family members in *P. multocida*‐induced pyroptosis merits consideration. While our data identify GSDMD as principal executioner in this context, emerging evidence indicates that distinct pathogens can engage alternative gasdermins to modulate host cell death pathways. For example, *Yersinia* species have been reported to trigger pyroptosis via GSDME activation, whereas *Streptococcus pyogenes* secretes the cysteine protease SpeB to directly cleave and activate GSDMA [37, 38]. Although the contribution of these alternative gasdermins (such as GSDME or GSDMA) to *P. multocida* pathogenesis has not yet been investigated, their potential involvement cannot be excluded and represents an important direction for future studies.

Clinically, the correlation between GSDMD activation and lung injury severity underscores its biomarker potential. While pyroptosis can augment pathogen clearance via cytokine release, excessive activation may precipitate cytokine storms and systemic inflammation. Our in vivo data confirm that GSDMD deficiency mitigates tissue injury and inflammation but compromises bacterial control, highlighting the need for a finely tuned therapeutic approach. Similar translational insights have been leveraged in the pathogenesis of cancer and inflammation, where GSDMD is not always protective against infections; instead, it may exacerbate infections and pathological inflammation by potentially triggering a cytokine storm [39]. Mechanistically, GSDMD mediates pathogenicity in this context by driving a maladaptive host immune response, where excessive pore formation leads to lytic cell death and a lethal cytokine storm that causes multiple organ failure. To better elucidate this pathogenicity, it is crucial to compare *P. multocida* with other bacterial models. Consistent with *E. coli*‐induced sepsis, GSDMD mediates pathogenicity in *P. multocida* infection primarily by driving a maladaptive host immune response—specifically, a lethal cytokine storm that outweighs the benefit of bacterial clearance [28]. However, a distinct feature of *P. multocida* appears to be its potential exploitation of GSDMD for initial invasion, as indicated by our adhesion assays. This contrasts with pathogens like *Salmonella*, where GSDMD functions strictly as a downstream clearance mechanism, highlighting a novel pathogen‐specific subversion strategy [40]. Further studies showed that GSDMD knockout mice infected with *P. multocida* had alleviated lung pathological damage and significantly reduced IL‐1β secretion, but this was accompanied by a higher bacterial load in lung tissue. This phenomenon may be attributed to the fact that GSDMD‐mediated pyroptosis in WT mice enhances the immune response by releasing inflammatory cytokines (such as IL‐1β), thereby restricting bacterial dissemination [41]. Integrating GSDMD inhibition into veterinary protocols could mitigate *P. multocida* ‐associated septic shock in livestock, a pressing need given its economic impact on agriculture [42].

In conclusion, this study advances our understanding of *P. multocida*’s immune subversion tactics while highlighting GSDMD as a pivotal therapeutic node. Future work should prioritize identifying *P. multocida* ‐specific pyroptosis inducers, exploring cross talk between gasdermins, and validating combinatorial therapies in preclinical models. Such efforts will not only refine treatment paradigms for *P. multocida* infections but also contribute to the broader field of pyroptosis‐targeted immunomodulation.

## Author Contributions

Qingqing Yang, Xin Shen, Wei Wang, and Jiajia Zheng performed the experiments. Yi Lu and Jinrong Ran helped analyze the data. Xuefeng Cao and Rendong Fang supervised the study and designed the experiments. Qingqing Yang, Xuefeng Cao, and Rendong Fang drafted the manuscript.

## Funding

This study was supported by the National Natural Science Foundation of China (Grants 32172850, 32473027, and 32503042), National Key Research and Development Program of China (Grant 2021YFD1800800), the New Chongqing‐Young Elite Program (Grant CSTB2024YCJH‐KYXM0078), Natural Science Foundation of Chongqing, China (Grant CSTB2025NSCQ‐GPX0497), Chongqing Modern Agricultural Industry Technology System (Grant CQMAITS202512), and the Southwest University graduate students research innovation project (Grant SWUS24194).

## Disclosure

All authors have read and agreed to the published version of the manuscript.

## Conflicts of Interest

The authors declare no conflicts of interest.

## Supporting Information

Additional supporting information can be found online in the Supporting Information section.

## Supporting information


**Supporting Information 1** Figure S1: Disulfiram treatment attenuates PmCQ2‐induced pyroptotic cell damage. Representative confocal microscopy images of PECs pretreated with disulfiram and then infected with PmCQ2 (A). Cells were stained with DAPI (blue, nuclei) and an anti‐GSDMD antibody (red).

**Supporting Information 2** Figure S2: NAC inhibits ROS production and alleviates pyroptosis induced by PmCQ2. Cell death was assessed by PI staining in cells pretreated with NAC and infected with PmCQ2 (A). In addition, western blot analysis showing the activation levels of key pyroptosis‐related proteins, including GSDMD‐N and caspase‐1 (B). And intracellular ROS levels were measured using the fluorescent probe DHE (C).

## Data Availability

All data are available within the article and from the corresponding author upon reasonable request.
