# Supplementary figures and images for: Mechanism of GSDMD in the Pathogenesis of Pasteurella multocida PmCQ2
Source: Transbound Emerg Dis. 2026 Feb 11;2026:4436022. doi: 10.1155/tbed/4436022 (PMC12894785; doi:10.1155/tbed/4436022)

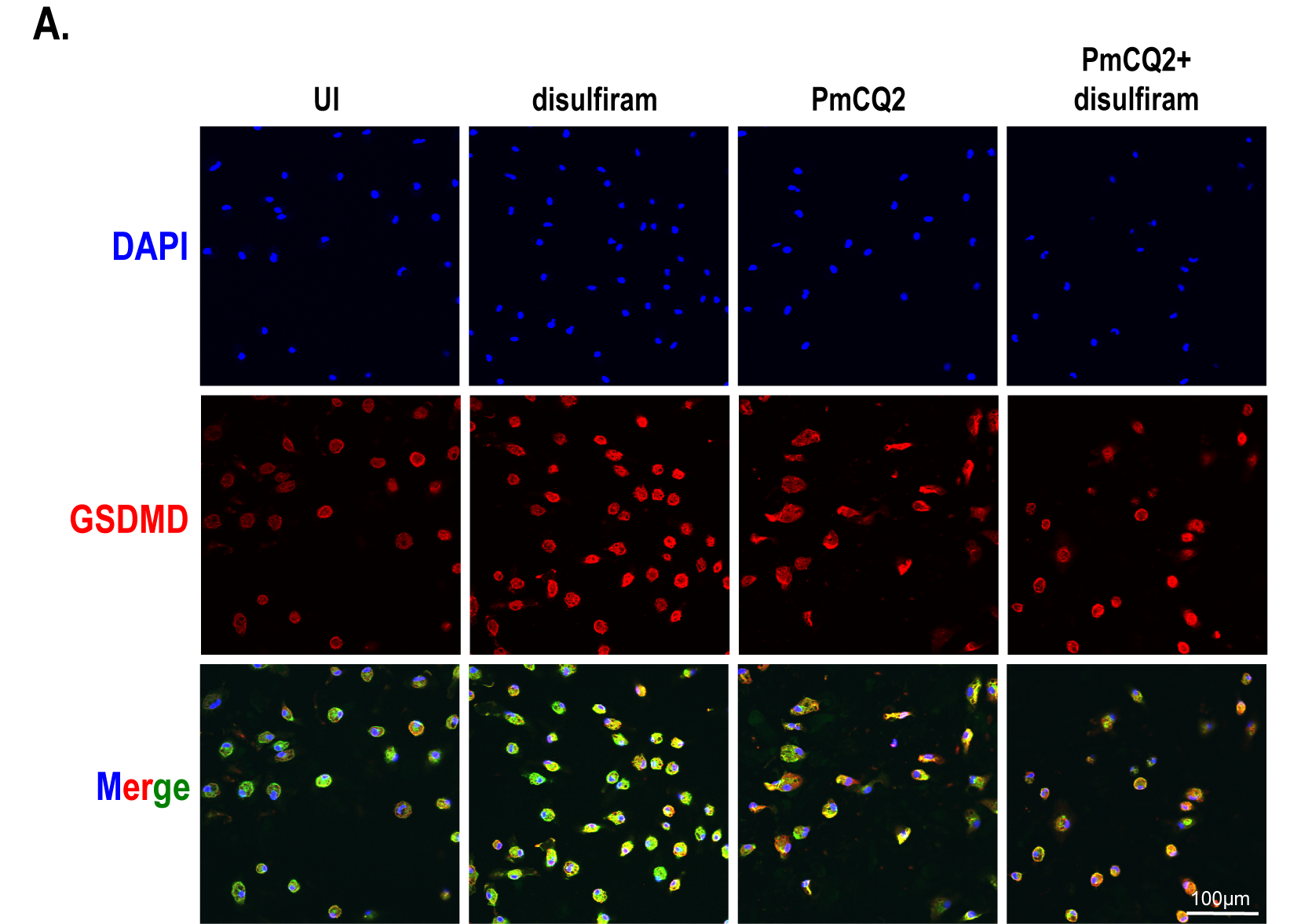

Supplement: Supplementary file 1 — Supporting Information 1 Figure S1: Disulfiram treatment attenuates PmCQ2‐induced pyroptotic cell damage. Representative confocal microscopy images of PECs pretreated with disulfiram and then infected with PmCQ2 (A). Cells were stained with DAPI (blue, nuclei) and an anti‐GSDMD antibody (red). [file TBED-2026-4436022-s001.docx]

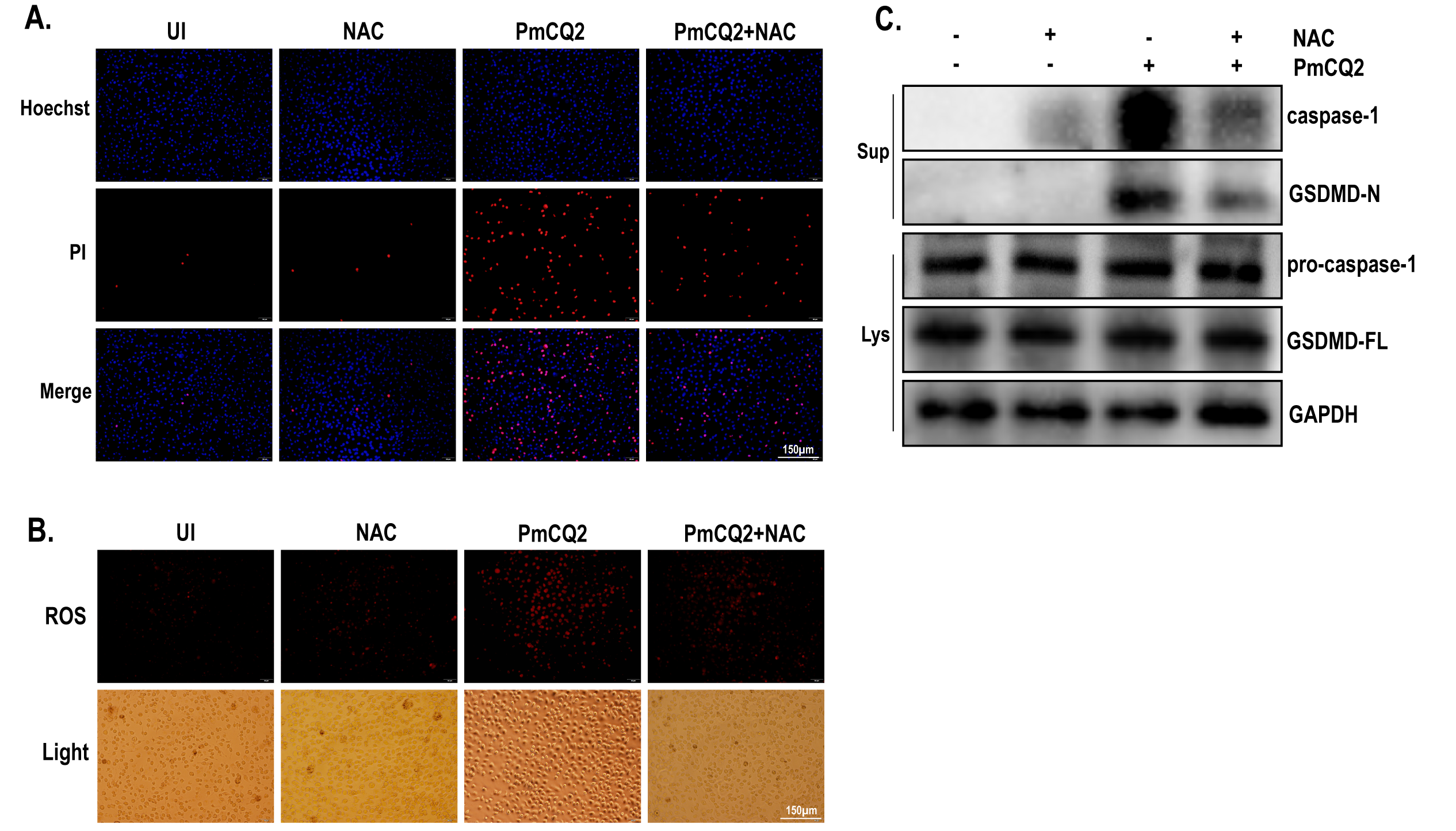

Supplement: Supplementary file 2 — Supporting Information 2 Figure S2: NAC inhibits ROS production and alleviates pyroptosis induced by PmCQ2. Cell death was assessed by PI staining in cells pretreated with NAC and infected with PmCQ2 (A). In addition, western blot analysis showing the activation levels of key pyroptosis‐related proteins, including GSDMD‐N and caspase‐1 (B). And intracellular ROS levels were measured using the fluorescent probe DHE (C). [file TBED-2026-4436022-s002.docx]
